# Correlation between perioperative immunological changes and the onset of surgical site infection after surgery for scoliosis: a retrospective cohort study

**DOI:** 10.1186/s40981-020-00327-6

**Published:** 2020-03-10

**Authors:** Keika Mukaihara, Maiko Hasegawa-Moriyama, Yuichi Kanmura

**Affiliations:** 1 Department of Anesthesiology and Critical Care Medicine, Graduate School of Medical and Dental Sciences, Kagoshima University, 8-35-1 Sakuragaoka, Kagoshima, 890-8520 Japan; 2 Present Address: Department of Operating Suite, Shinshu University Hospital, 3-1-1 Asahi, Matsumoto, Nagano, 390-8621 Japan

**Keywords:** Surgical site infection, Scoliosis, C-reactive protein

To the editor,

Surgical site infection (SSI) is a major complication of spinal surgery involving implants. Although immunosuppressive effects of anesthesia including opioids have been implicated [1], whether postoperative infection can be predicted in the early period after surgery remains to be fully elucidated. Therefore, we investigated whether a correlation exists between perioperative immunological changes and the onset of SSI.

We retrospectively reviewed 127 patients who underwent surgery for scoliosis at Kagoshima University Hospital from January 2012 to December 2017. In all patients, anesthesia was induced with propofol, remifentanil, and rocuronium. Anesthesia was maintained with continuous intravenous target-controlled infusion of propofol with an effect-site concentration of 2.5 to 4.0 μg/mL for patients ≥ 16 years old or continuous infusion at a rate of 4.0 to 6.0 mg/kg/h for patients < 16 years old to maintain the bispectral index at 40 to 60. During surgery, remifentanil was administered at 0.2 to 1.0 μg/kg/min. Rocuronium was not used to measure motor evoked potentials during the operation. After the end of surgery, sedation with dexmedetomidine (0.4–0.7 g/kg/h) and propofol (3.0–4.0 g/kg/h) was continued until arrival in the intensive care unit.

SSI occurred in 7 of 127 patients (5.5%). The onset of SSI differed among the patients, ranging from postoperative day (POD) 8 to POD 77. The total use of remifentanil was not different between patients with and without SSI. The preoperative C-reactive protein (CRP) level was significantly higher in patients with than without SSI [median (interquartile range), 0.5 (0.09–0.63) vs. 0.03 (0.02–0.15) mg/dL, respectively; *P* = 0.026]. The immunological parameters such as the neutrophil count, lymphocyte count, and N/L ratio were not different between patients with and without SSI immediately after the end of anesthesia although an elevated N/L ratio is reportedly correlated with the incidence of SSI after posterior lumbar instrumentation surgery [2]. Similarly, the plasma CRP level on both POD 7 and POD 14 but not POD1 was significantly higher in patients with than without SSI [POD 1 4.41 (1.77–6.24) vs. 4.11 (2.46–5.61) mg/dL, respectively, *P* = 0.979; POD 7 3.55 (2.82–11.68) vs. 1.56 (0.84–2.62) mg/dL, respectively, *P* < 0.01; and POD 14 7.29 (1.14–9.74) vs. 0.31 (0.14–0.90) mg/dL, respectively, *P* < 0.01]. This is consistent with a previous report showing that the CRP level on POD 7 is a reliable marker for SSI following instrumented spinal fusion [3]. Postoperatively, the duration of intensive care unit stay was not different between the groups. However, the postoperative duration of fentanyl infusion was significantly longer in patients with than without SSI [12 (4.5–17) vs. 2 (2–5) days, respectively; *P* = 0.015], possibly because of postoperative pain due to infection.

In conclusion, immunological parameters immediately after the end of anesthesia were not different between patients with and without SSI. The correlation between perioperative opioid use and the incidence of SSI was not clear. The parameter directly reflecting the onset of SSI might be CRP level after POD7.

## Data Availability

The datasets analyzed during the current study are available from the corresponding author on reasonable request.

## Abbreviations

CRP: C-reactive protein
N/L ratio: Neutrophil/lymphocyte ratio
POD: Postoperative days
SSI: Surgical site infections
